# Founder mutations in Tunisia: implications for diagnosis in North Africa and Middle East

**DOI:** 10.1186/1750-1172-7-52

**Published:** 2012-08-21

**Authors:** Lilia Romdhane, Rym Kefi, Hela Azaiez, Nizar Ben Halim, Koussay Dellagi, Sonia Abdelhak

**Affiliations:** 1 Laboratory of Biomedical Genomics and Oncogenetics, Institut Pasteur de Tunis, BP 74, 13 Place Pasteur, Tunis 1002, Tunisia; 2 GIS CRVOI, Centre de Recherche et de Veille sur les Maladies Emergentes dans l'Océan Indien, Saint-Denis, La Réunion; 3 Institut de Recherche pour le Développement, PB 50172, 97492, Ste Clotilde, France

**Keywords:** Rare genetic disorders, Founder mutations, Common haplotype, Diagnosis, Mutation screening, Tunisia, North Africa, Middle East, Ethnicity

## Abstract

**Background:**

Tunisia is a North African country of 10 million inhabitants. The native background population is Berber. However, throughout its history, Tunisia has been the site of invasions and migratory waves of allogenic populations and ethnic groups such as Phoenicians, Romans, Vandals, Arabs, Ottomans and French. Like neighbouring and Middle Eastern countries, the Tunisian population shows a relatively high rate of consanguinity and endogamy that favor expression of recessive genetic disorders at relatively high rates. Many factors could contribute to the recurrence of monogenic morbid trait expression. Among them, founder mutations that arise in one ancestral individual and diffuse through generations in isolated communities.

**Method:**

We report here on founder mutations in the Tunisian population by a systematic review of all available data from PubMed, other sources of the scientific literature as well as unpublished data from our research laboratory.

**Results:**

We identified two different classes of founder mutations. The first includes founder mutations so far reported only among Tunisians that are responsible for 30 genetic diseases. The second group represents founder haplotypes described in 51 inherited conditions that occur among Tunisians and are also shared with other North African and Middle Eastern countries. Several heavily disabilitating diseases are caused by recessive founder mutations. They include, among others, neuromuscular diseases such as congenital muscular dystrophy and spastic paraglegia and also severe genodermatoses such as dystrophic *epidermolysis bullosa* and *xeroderma pigmentosa*.

**Conclusion:**

This report provides informations on founder mutations for 73 genetic diseases either specific to Tunisians or shared by other populations. Taking into account the relatively high number and frequency of genetic diseases in the region and the limited resources, screening for these founder mutations should provide a rapid and cost effective tool for molecular diagnosis. Indeed, our report should help designing appropriate measures for carrier screening, better evaluation of diseases burden and setting up of preventive measures at the regional level.

## Background

More than 340 genetic diseases have been identified in the Tunisian population. In a previous study, we have reported on the spectrum of these disorders. The genetic aetiologies are known for only 50% and are caused by at least one mutation [1]. Due to the relatively high rate of endogamy and consanguinity, over 60% of genetic diseases in Tunisia are autosomal recessive [1]. This socio-cultural feature is shared by several other countries mainly in North Africa and Middle East. Individuals whose parents share a common ancestor are more likely to have inherited two copies of the same single allele (identical by descent). The flanking genomic sequence is usually inherited identical by descent, as well. Thus, consanguineous off springs of mating between relatives are more often “homozygous by descent” [2]. The probability that the consanguineous child of union between relatives will be autozygous for a particular allele is calculated by the coefficient of inbreeding noted “F” [3]. Many genetic disorders are due to mutations that could be traced back to a founder whose existence can be inferred from the particular chromosomal background on which the mutation arose [4]. Founder mutations are of particular interest because they provide a rapid tool for molecular diagnosis of some genetic disorders. Indeed, testing for one or a few prevalent founder mutations is more efficient than for many rare mutations [5]. Targeted screening of ethnically restricted disease mutations in the appropriate population subgroups has also demonstrated its efficiency in disease prevention [6]. This enhanced the importance of the population history which can have a serious impact on medical genetics [4,7].

The following is an overview of the Tunisian population structure and history.

### Tunisian population origins

**Figure 1:**
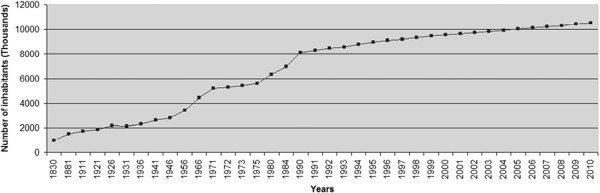
**Tunisian population growth [**[8]**,**[9]**,**[16]**].**

Tunisia has approximately 10 million inhabitants. It is three times as much as in 1956 at the end of the French protectorate, and twice as much as in the seventies (Figure 1) [8,9]. This growth is mainly attributed to the decrease of infantile mortality rates and the improvement of life expectancy; which are indicators of improvements in health status and medical management [9]. African, Near Eastern and European influences have shaped the Tunisian cultural identity. The overwhelming majority of the population is Muslim (98%) . Christian and Jewish communities also contribute to the Tunisian cultural diversity [10]. Like the other North African and Middle Eastern countries, Tunisia has kept the consanguineous and endogamous marriages as a social habit. Indeed, the frequencies of unions between relatives range from 20.1% to 39.33% [11-14] and those of endogamous marriages could reach more than 96% in some specific groups like those of Douiret in Southern Tunisia [Ben Halim, Unpublished data]. The Tunisian family structure is generally extended and often associated with inbreeding. Tunisia is classified as a middle-income country [15].

### Prehistoric background

Tunisia has been inhabited since the Paleolithic period. Remainders of Acheulean industry (700,000 to 100,000 years BCE) have been discovered on several sites in the layer of Sidi Zin in the north west, Gafsa and Redayef in the south. The 100,000 last years of Paleolithic were characterized by the presence of *Homo sapiens archaic* contemporary of *Homo neanderthalensis* in Europe. Between 60,000 and 20,000 years BCE, the Mediterranean coast stretching over from Cap Bon to Monastir was occupied by *Homo sapiens sapiens.* In the Epipaleolithic period (23,000 years to 10,000 years BCE), the Mechta El Arbi Man, *Homo sapiens sapiens* specific to North Africa succeeded to the Aterian population. Around 8,000 years BCE, the Capsian civilization first identified in the eponymous city of Gafsa covered a broad area of Central and Southern Tunisia and could be considered as the ancestor of the Berber population. Berbers occupied the North African region including Tunisia. They have been for long self-identified as “Imazighen” which means “free people” [17,18].

### Major historical events

The Punic era initiated with the arrival of Phoenician traders from the eastern Mediterranean Sea and was marked by the founding of the City of Carthage on 814 BC (present Tunis). For many centuries, the Punic civilization either displaced the native Berbers to the city periphery or integrated them. Phoenicians expanded their dominance to the western Mediterranean and had settled in what are now Sardinia, Sicily, Spain, Morocco and Algeria. The emergence of the Roman Republic led to sustained rivalry with the more anciently established Carthage for dominion over the western Mediterranean. Carthage lost its influence over the Berbers leading to the defeat of Carthage during the Third Punic War (149–146 BC), therefore, marking the end of the Phoenician reign. Tunisia became the Roman Province of Africa. After the dissolution of the Roman Empire during the 5^th^ century, the Germanic nation of the Wisigoths migrating downward to south Western Europe (the so called Vandals) took control of the coastal part of North Africa for nearly a hundred years. The Byzantine Empire re-established its dominion in 533 AD, which lasted till the Islamic conquest in the 7^th^ century. This era started with the arrival of Arabs from the Arab Peninsula who brought their language, their culture and Islam [19]. They founded Kairouan in 670 AD, the first Arab-Muslim city in “Ifriqya” (nowadays: Tunisia, Eastern Algeria and Western Libya). Successive Arab dynasties were established in the region. The 11^th^ century was marked by a breaking event: the invasion of Bedouins of Banu Hilal and Banu Sulaym originating from the Banu’Amir tribe from the southwest Arabia. These Arab newcomers (up to 200,000 individuals) represented the second large Arab immigration into Ifriqiya [19].

In 1135 AD, Ifriqiya was attacked by Normands and Sicilians who occupied four coastal cities of Tunisia. They were then expelled under the reign of the Almohad dynasty (1147–1269 AD) [19]. The arrival of Béni Soulaim in Tunisia had accelerated the administrative desagregation of the country and the dissolution of the Almohad dynasty [19]. Under the Hafsids (1230–1574 AD), the Black Death has caused extensive devastation from Tunis to Tanger. Since then it had hit every 10 years in addition to droughts and starvations [20,21]. Spain had occupied a series of positions in North Africa including Tunis (1534 AD) until their incorporation in the Ottoman Empire. The population size of the region had evolved from 4.7 millions around 1500 AD, to 5.4 millions in 1620 AD after the mass migration of hundreds of thousands of Morrish Andalusian Muslims and Jews expelled from Spain to North Africa [19-21]. The 16^th^ and the 17^th^ centuries were characterised by successive epidemic waves, cricket invasions and starvations leading to severe human losss that reached hundreds of thousands [20,21]. The beginning of the 18^th^ century was hallmarked by the decline of the Ottoman Empire and the reemergence of plague that hit the entire Maghreb until the first half of the 19^th^ century [20]. The Tunisian population growth was periodically slowed by successive and prolonged epidemics of plague, cholera and typhus during the 19^th^ century as nearly one thousand individuals had died in the city of Tunis in 1918 [21]. In 1881, French forces established a protectorate. In 1936, the European population (French, Italian and Maltese) in Tunisia was of more than 213 thousands inhabitants [16]. The French protectorate ended in 1956 AD [19].

### Tunisian Jews

A Jewish minority has existed in Tunisia since the Roman times. In the 7th century, the Jewish population in Tunisia largely increased due to Spanish immigrants fleeing the persecutions of the Visigoth king. They fled to Morocco and settled in the Byzantine cities. These settlers, according to the Arab historians, mixed with the Berber population and converted many tribes [22]. Later, during the Islamic expansion they continued to profess Judaism until the reign of the Idrisid dynasty in Western Maghreb (789–985 AD) though they could maintain their particularism thanks to the permissive Islamic rule of Dhimma. Molecular studies based on the analysis of mitochondrial DNA and Y-chromosome polymorphisms showed a negligible or low level of admixture of the Jewish community with Arab and Berber populations in Tunisia [23,24]. As a conclusion, the genetic landscape of the Tunisian population has been shaped by successive invasions and migratory flows through its history. Epidemics, droughts and plagues have also impacted the Tunisian population [20,21]. The new-comers and the local Berbers intermarried and mixed to some extent, although, some Berber groups like the Berbers of Douiret in the South and those of “El Gallala” in the island of Jerba are known to have poorly mixed with new-comers and are still Berber speaking and highly endogamous. Genetic investigations based on mitochondrial DNA analysis of current populations from Berber villages in Tunisia showed the absence of a common genetic Berber profile. Genetic differentiation was observed between Berbers from different localities (Sejnane, Takrouna, Kesra and Jerba Island) and between Berbers and Arabs (Zriba and Jerba Island) as well as among Jews (Jerba Island) [25-27]. This genetic heterogeneity between these current small groups is due essentially to founder effects and could also be due to their limited size and the variation of Sub-Saharan African traces detected in their gene pool. Nevertheless, when comparing samples from neighboring populations, the general ancient genetic profile of the native North Africans –the Berbers- appears not to be very different from that of the present-day North African populations despite some admixture with other people, particularly Arabs of little genetic contribution. This suggests that the populations of the Maghreb seem to share a substantial genetic background [28-30].

In a country with limited resources and with other public health and research priorities, the rare genetic diseases remain neglected. As no complete report on founder mutations in the Tunisian population was performed, we have collected data on these mutations in the region and discussed their usefulness for the design of cost effective diagnosis tool in the Tunisian, North African and the Middle Eastern populations.

## Methods

To identify founder mutations among the Tunisian population, we searched the databases Pubmed and OMIM through March 2012 using combinations of the following key-words “founder, mutation, Tunisia, North Africa, Middle East, common, haplotype”. Furthermore, references cited in the published papers were examined until no further study was identified. Publications in English and French provided essential information. Abstracts from meeting proceedings were also retrieved. Geographic and population distributions of mutation were further checked by querying also available population and locus-specific databases. We present information about the timing of occurrence of these mutations and their contemporary geographic distribution in Tunisia when it is known. Mutation description according to the HGVS nomenclature was checked with the Mutalyzer version 2.0 β-4 software [31]. Some of them are reported as mentioned in the original text. When available, data on number of affected individuals and families or on carrier frequency are provided.

## Results

In a recent study of the genetic disorders in Tunisia, over 340 genetic diseases were identified [1]. For 73 genetic conditions, one or several founder or likely founder mutations were reported. The classification according to the mode of transmission revealed that 63 (86%) are autosomal recessive, 8 (11%) are autosomal dominant and 2 (3%) are X-linked.

All the 73 reported diseases originated from at least one founder or one likely founder mutation. A founder mutation being a mutation that arose in a common ancestor, the same mutation is found in related individuals who share the same haplotype; it can be of low or high frequency in the population [32]. We have assigned diseases due to founder mutations to three main categories according to the geographic distribution:

(1) Diseases with Tunisian specific founder mutations; when the mutation was identified only in the Tunisian population among unrelated individuals (Additional file 1).

(2) Diseases with mutations shared with North African and Middle Eastern populations that are likely founder, and in some rare cases, mutations were shared with other populations mostly from Europe, the Mediterranean Basin and America (Additional file 2).

(3) Diseases with both Tunisian specific and shared alleles with other populations (Additional file 3).

When common origin is not demonstrated on the basis of haplotype sharing, recurrent mutation could not be ruled out.

### Tunisian specific founder mutations

**Figure 2:**
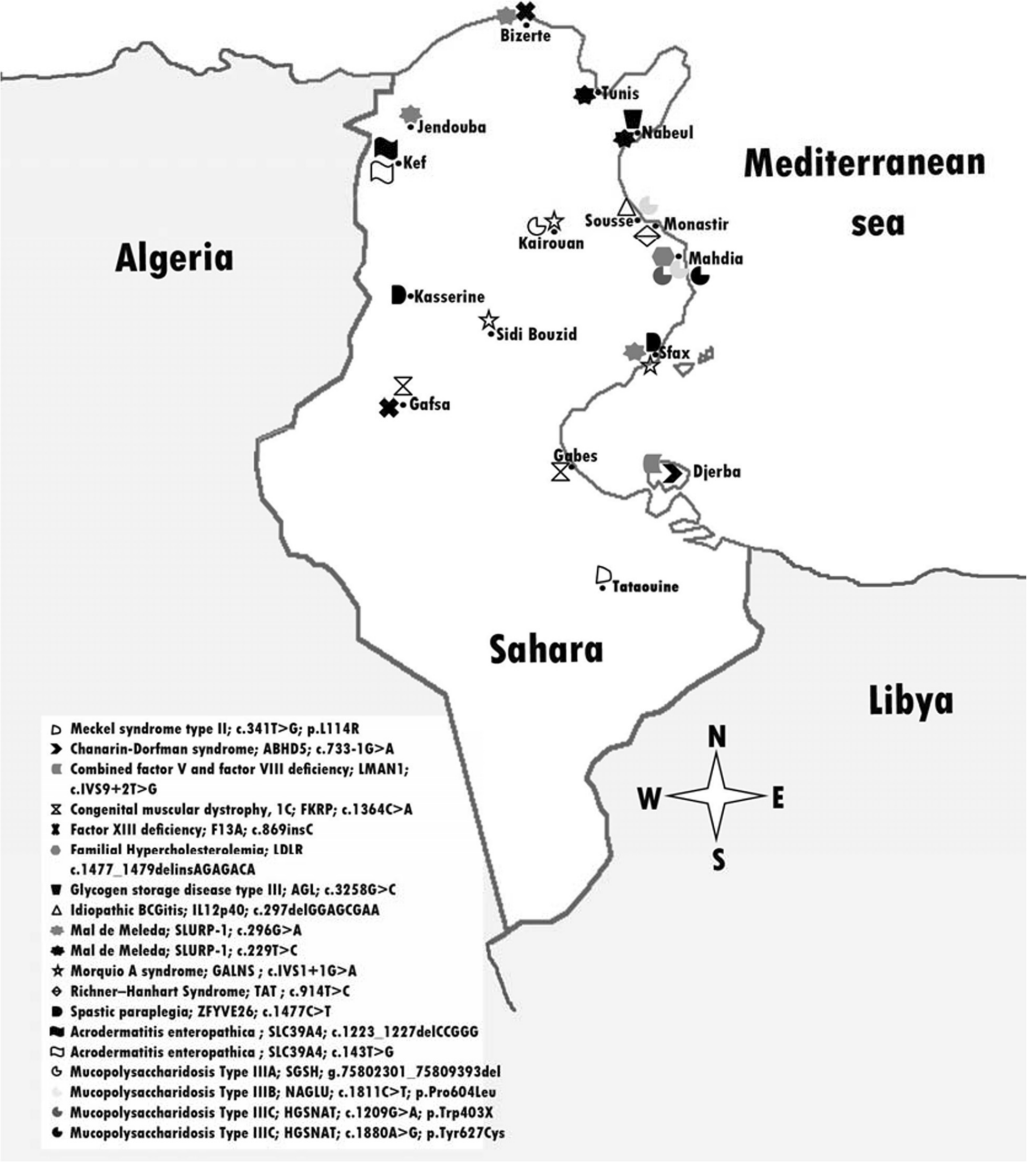
Geographic distribution of Tunisian specific founder mutations.

In the Tunisian population, 22 genetic diseases are due to Tunisian specific founder alleles and likely founder alleles whereas 8 genetic diseases can be due to either Tunisian-specific alleles or non-specific alleles shared with other North African or Middle Eastern populations. Sixty two percent (21/34) mutations are due to common ancestors on the basis of shared haplotypes (Additional file 1, Additional file 3 and Figure 2). For the majority of these Tunisian founder mutations, geographic distribution is limited to a region of the country like mutations responsible for congenital muscular dystrophy 1C [Louhichi N, personal communication] and spastic paraplegia 15 encountered in Southern Tunisia [Boukhris A, personal communication]. The p.L273P founder mutation affecting the *TAT* gene that leads to Richner–Hanhart Syndrome is restricted to the region of Monastir [Charfeddine C, personal communication]. The p.Ser493ArgfsX11 mutation in *LDLR* gene causing familial hypercholesterolemia is limited to the village of Souassi [33] and the deletion in *IL12p40* gene of disseminated BCGitis is restricted to the village of Akouda [34] and several other examples (Figure 2).

Some other founder mutations are specific to an ethnic and religious group such as the c.890_893del mutation in *FANCA* gene that underlies Fanconi anemia specifically among the Tunisian Jews [35]. They could be also specific to both an ethnic group and a geographic region. This is illustrated by the Jewish community living on the Island of Jerba who is affected with combined factor V and factor VIII deficiency due to the c.IVS9+2T>G mutation in *LMAN1* gene [36]*.*

### Allele sharing with other North African and Middle Eastern populations

The Tunisian population shares founder mutations with other North African and Middle Eastern populations for 43 inherited conditions whereas 8 genetic diseases are due to either shared or specific alleles (Additional file 2, Additional file 3). Common haplotypes described in the Tunisian population could also be reported in restricted regions of North Africa. Founder chromosomal segments described in Tunisian patients with Meckel syndrome, mal de Meleda (MDM), sickle cell anemia and *Xeroderma pigmentosum* (XP) group A are identical to those described in Algerian affected individuals [37-40]. In addition, the founder mutations leading to adenomatous polyposis of the colon and the hepatocerebral mitochondrial DNA depletion syndrome are reported on the same haplotypes only among Tunisian and Moroccan patients [41,42].

Founder mutations could be shared in some countries of the Maghreb (defined today as Morroco, Tunisia, Libya, Mauritania and Algeria). In Tunisian, Algerian and Moroccan patients, autosomal recessive non syndromic optic atrophy and Bare lymphocyte syndrome are caused by the p.R2034X and 752delG-25 founder mutations, respectively [43,44]. Identical haplotypes bearing founder mutations are observed for congenital myasthenia syndrome and XP group C in four North African countries [45,46].

Founder mutations observed in Tunisia were also reported in populations from the Middle East such as the splice site mutation in the *CA II* gene responsible for carbonic anhydrase II deficiency in the Arabic peninsula [47] and the p.Q357R molecular defect in the *UGT1A1* gene of the Crigler-Najjar type I syndrome in the Kuwaiti population [48]. In other cases, founder mutations are dispersed around the Mediterranean basin as illustrated by the c.744delA mutation in the *TTPA* gene of ataxia with isolated vitamin E deficiency [49] and the splice site defects in megaloblastic anemia 1 [50] and triple-A syndrome [51]. In other examples, the founder haplotypes are shared with populations from Europe and America, as for the Parkinson disease [52].

Some other founder mutations are reported in specific ethnic and religious groups mainly Jews from North Africa. The p.R35X founder mutation, responsible for ataxia-telangiectasia, is mainly reported in Jews from Tunisia and Morocco [53]. Common haplotype bearing the large CGG repeats on the *FMR1* gene, responsible for fragile X, is observed among Tunisian and Sephardic Jewish patients and among Israeli Arabs as well [54]. A common founder mutation responsible for cutaneaous malignant melanoma was also reported only among Jewish patients from Tunisia, Morocco, Spain and France [55].

A same mutation could have arisen on different haplotypes. This could be explained by independent mutational events that could have occurred and have been introduced into the population by different founders during migrations and invasions. This is a possible hypothesis if we take into consideration the rich history of the Tunisian population. This is the case for mutations underlaying beta thalassemia [40], sickle cell anemia [40], familial Mediterranean fever [56], G6PD deficiency [57], 21 hydroxylase deficiency [58,59], limb-girdle muscular dystrophy type 2C (LGMD2C) [60] and non syndromic hearing loss [61] that show a large distribution arround the Mediterranean basin.

### Diseases with both Tunisian specific and shared alleles

For the same disease and/or the same causing gene, some mutations could be specific to the Tunisian population and others are shared by other populations (Additional file 3). Eight genetic diseases belong to this category. This is the case for 11-β hydroxylase deficiency [62,63], hereditary breast and ovarian cancer [64,65], cystic fibrosis [66,67], glycogen storage disease type III [68,69], Leber congenital amaurosis [70], leukocyte adhesion deficiency [71,72] and MDM [Charfeddine C, personal communciation, 37,73]. For some founder mutations, the situation is relatively complex. Indeed, the p.C99Y mutation responsible for MDM is distributed in various geographic areas of the country, while the p.C77R mutation is restricted to the North. On the other hand, the c.82delT mutation is widely encountered in populations from the Mediterranean basin and also in Scottish and Croatian ones [37,73].

Multiple founder effects are encountered for 4 inherited diseases caused by more than one founder mutation (Additional file 3). This phenomenon could affect the same gene. This is illustrated by the examples of hereditary breast and ovarian cancer [64,65], leukocyte adhesion deficiency [71,72] and MDM [Charfeddine C, personal communciation,73]. Another situation is encountered in genetically heterogeneous diseases with founder mutations in different underlying genes as is the case with Bardet-Biedl syndrome where two founder mutations affecting both *BBS2* and *BBS8* genes were reported [74] (Additional file 1).

## Discussion

More than three hundred genetic disorders were reported in Tunisia [1]. Among them, 73 inherited diseases were likely due to a founder effect representing 42% of all the disorders with identified molecular defect described in Tunisian patients [1]. It is noteworthy that for half of the diseases reported in Tunisia, the genetic basis is still unknown; consequently, the proportion of founder mutations might be even larger and will expand with further identification of disease causing mutations. The high proportion of recessive founder alleles (86%) points out the role of endogamy and consanguinity as a major cause of the high prevalence of the diseases they cause.

Allelic heterogeneity is also noticed for 23 genetic diseases out of the 73 reported in this study. Two situations depict the fact that multiple mutations could affect the same gene responsible of a single genetic disease in a small geographic area or isolated population. In the first situation, one mutation is predominant thus consolidating the hypothesis of founder effect and genetic drift. In addition to this frequent mutation, rare or private deleterious variants are also observed and could be new mutations or introduced by migrations [75,76]. This case is illustrated by the LGMD2C which is caused by three mutations one of which, the c.521delT is by far the most predominant [60,77,78]. The same picture is also shown for the major founder mutation p.R228X in *xeroderma pigmentosum* group A. Another private mutation was recently reported in one family [39,79]. In the second observation, more than one frequent mutation is responsible for the genetic disease. The most illustrating examples are beta-thalassemia [40], cystic fibrosis [66] and familial Mediterranean fever [80]. A possible explanation for this observation is the selective advantage of heterozygous in the case of recessive disorders [75,81]. The existence of multiple founder mutations affecting the same gene exclusively in the Tunisian population as in the case of Acrodermatitis enteropathica [82] and MDM [73] could be explained by an autochtonous mutational event. As these diseases have not yet been investigated in the neighboring countries, specificity of the mutations to Tunisian population should be taken cautiously.

Some mutations had a very limited geographical distribution in Tunisia like the p.G1483D and p.R1763X mutations in the *COL7A1* gene that are encountered specifically in the region of Sidi Bouzid and Kasserine, respectively [Unpublished data]. It is noteworthy that these mutations have been reported among Kuwaiti patients [83]. These mutations likely arose in the Middle East and were introduced in Tunisia following Banu Hilal invasions that settled in the Maghreb in the eleventh century. Several other mutations like those responsible for carbonic anhydrase and Crigler Najjar disease were introduced similarly [47,48]. The extent of geographic repartition of a founder mutation could give insights into its history and age. Older founder mutations tend to be widely distributed; mainly due to migration. This is the case for c.35delG mutation in *GJB2* that causes deafness and is widely spread in Europe and around the Mediterranean basin. As haplotype data suggested, it arose from a single mutational event about 10,000 years ago somewhere in the Mediterranean region or in the Middle East. Then it spread throughout Europe along the two Neolithic population movement routes [84-86]. Comparing frequency data of *HBB* gene mutations such as the c.118C > T (also described as the codon 39 C > T mutation) and c.IVS1-110G>A substitutions within the different parts of the Maghreb region reveals important differences. The c.IVS1-110G>A mutation is predominant in the western part of Tunisia and in northern Algeria [40]. This fact is attributed to an introduction by Ottomans during their dominance over this region that does not reach the Moroccan territory, thus explaining its rarity in Morocco. However, the codon 39 C > T mutation is largely predominant in Tunisia, Morocco and north-western Algeria. It is thought to have an occidental origin and that it was introduced in the region during the Roman period through Italy [40]. The c.1070A > G mutation in *UGT1A1* gene could be considered as the most recent as it arose 32 generations ago [48]. The age of the founder event was estimated to approximately 700 years for c.1293insG mutation in *CHRNE* gene [45], 80 generations for the autosomal non syndromic optic atrophy (2400 years) [43] and calculated to have arisen in the second half of the 13^th^ century for Creutzefeldt-Jacob disease [87].

Knowledge of the genetic disease features and of the spectrum of their underlying likely founder molecular defect and their distribution among different groups of the Tunisian population could influence different aspects of clinical practice: differential diagnosis, design of diagnostic test, selection of the most suitable diagnostic test and result interpretation [88].

Application of a suitable molecular diagnostic test is useful for the diagnostic orientation especially for phenotypically homogenous but genetically heterogeneous diseases. Screening for the founder c.del521T mutation in *SGCG* gene may be at the top of diagnosis analysis as this form of severe childhood autosomal recessive muscular dystrophy (SCARMD) is the most frequent muscular dystrophy in Tunisia [89] and that most patients were homozygous for this mutation [60]. Though other forms of SCARMD were reported in Tunisia, detection of this mutation should be considered as the first diagnostic step of the disease.

Disease particularities in the local population must be taken into consideration when diagnostic tests are developed. In some cases when internationally agreed clinical diagnostic criteria are lacking and biochemical tests do not allow accurate diagnosis in all cases, a molecular test is more suitable and appropriate [90]. Familial hypercholesterolemia (FH), a dominantly inherited and highly atherogenic condition due to a dysfunction of the LDLR, is frequent in Tunisia since carrier frequency is of 1/165 [33,91]. Its diagnosis traditionally based on elevated total cholesterol levels is challenging since FH patients could present a mixed hyperlipidemia phenotype with elevation of both LDL-cholesterol and triglycerides [92,93] which is a feature of another inherited lipid abnormality; the familial combined hyperlipidemia [90,94,95]. Therefore, the “Souassi” founder mutation should be screened first for the molecular diagnosis of FH in Central Tunisia since it is the most frequent in that area [33]. Early diagnosis for FH is very important as there is good evidence that early cholesterol-lowering therapy will delay or even prevent coronary heart disease in these persons [95].

The identification of the most prevalent mutation in different ethnic groups opened the way for molecular diagnosis particularly for metabolic diseases for which the biochemical investigation is unavailable in the country. This was the case for glycogenosis type Ia. Indeed, in the Tunisian population, two mutations in the *G6PC* gene, p.R38C and p.R170Q, account for more than 90% of mutations [96]. Therefore, developing direct DNA mutation analysis of the *G6Pase* gene allowed rapid carrier testing and avoided performing a liver biopsy for the biochemical confirmation [96]. For such disease and for some others, establishing the molecular basis allows a safer and less invasive diagnostic test therefore making it more acceptable and bearable to patients and their families.

In the context of limited resources for mutation screening of diseases that have not been studied yet in Tunisia, prioritizing mutation screening by relying on deleterious gene variants already identified in neighbouring populations was demonstrated to be particularly efficient. The recurrent mutation p.V548AfsX25 affecting the *XPC* gene and leading to XP group C, a rare severe genodermatosis associated with skin tumours [97], was first described in Algerian and Moroccan patients at homozygous state [98]. In a recent study and in an attempt to elucidate the mutation spectrum of the *XPC* gene in the Tunisian population, the authors prioritized the screening of the p.V548AfsX25 mutation in 20 Tunisian XP group C patients [97]. They demonstrated the homogeneous *XPC* gene mutation spectrum and confirmed a founder effect in this population [97].

Knowledge of the patients‘ ethnic background, origin and/or religion is also important in making a diagnosis and must be taken into consideration especially in the case of migrations. For several decades and until now, an important migration is taking place from North African and Middle Eastern countries to industrialised ones for work and study purposes. Marriages of North African migrant population occur preferentially between people of the same national origin or between people from two different Arabic countries because of religious barriers. The religious isolation has also favoured consanguinity and endogamy thus maintaining rare disease haplotypes in the host land. For Hurler syndrome, the point molecular lesion p.P533R is known to be frequent in all the Maghrebian populations even in the Maghrebian immigrant population in France [99,100]. As p.P533R is a founder mutation in the Tunisian population, then a molecular diagnosis could be proposed for patients of Maghrebian origin. For congenital myasthenic syndrome, patients originating from four North African countries and living in France share the same haplotype bearing the c.1293insG mutation affecting the *CHRNE* gene [45]. The strong evidence for a single ancestral founder event in the North African population may have an important implication for diagnosis of congenital myasthenic syndrome in the immigrant Maghrebian and original populations. However, one should stress that because of fear of racial interpretation, some physicians feel uneasy to ask about the geographic origin of the patient. In addition, the patient’s family may prefer, for personal reasons and knowledgeability, not to reveal their origin or the genetic disease that affects their family [88].

## Conclusion

Founder mutations identified in the Tunisian population provide special advantages for DNA diagnosis and carrier screening programs due to a limited spectrum of disease mutations; consequently one or a reduced number of tests could have high diagnostic specificity and sensitivity. The distribution of founder mutations is the result of historical events and could shed light on them as well as on migratory movements during the last few centuries. Taking into account these events, our report on founder mutations provides a valuable decision making tool for the diagnosis and prevention of diseases in North Africa, Middle East and migrant populations living in Europe or elsewhere in the world. Targeted screening of ethnically restricted disease mutations in the appropriate population subgroups has demonstrated its efficiency in disease prevention [5].

## Abbreviations

FH: Familial hypercholesterolemia; MDM: Mal de Meleda; SCARMD: Severe childhood autosomal recessive muscular dystrophy; XP: *Xeroderma pigmentosum*.

## Competing interest

The authors declare that they have no competing interests.

## Authors’ contributions

LR collected, interpreted the data and wrote the manuscript. RK helped to draft the historic section of the manuscript. HA participated in the English revision of the manuscript. NBH helped to draw the map. KD has been involved in the study design and revising the manuscript critically for important intellectual content. SA conceived the study, participated in its design and coordination and helped to draft and to revise the manuscript. All authors read and approved the final manuscript.

## Supplementary Material

Additional file 1 **List of Tunisian specific founder mutations.** List of Tunisian specific founder and likely founder mutations. Precise geographic distributions in the country and ethnic groups are indicated when available. Click here for file

Additional file 2 **List of founder mutations shared with other North African and Middle Eastern countries.** Detailed list of founder and likely founder mutations in Tunisia and others populations from North Africa and Middle East are available in a comprehensive table. Precise geographic distributions in the country and ethnic groups are indicated when possible. Click here for file

Additional file 3 **List of diseases with both Tunisian specific and shared founder mutations.** Diseases with both Tunisian and shared mutations are available in a comprehensive table. Click here for file

## References

[B1] Romdhane L Abdelhak S Genetic diseases in the Tunisian population Am J Med Genet A 2011 155 238 267 10.1002/ajmg.a.33771 21204241

[B2] Teebi AS El-Shanti HI Consanguinity: implications for practice, research, and policy Lancet 2006 367 970 971 10.1016/S0140-6736(06)68406-7 16564347

[B3] Bridge PJ The Calculation of Genetic Risks – Worked examples in DNA diagnostics 1997 2 Johns Hopkins University Press, Baltimore

[B4] Zeegers MPA van Poppel F Vlietinck R Spruijt L Ostrer H Founder mutations among the Dutch Eur J Hum Genet 2004 12 591 600 10.1038/sj.ejhg.5201151 15010701

[B5] Pastinen T Perola M Ignatius J Sabatti C Tainola P Levander M Syvänen AC Peltonen L Dissecting a population genome for targeted screening of disease mutations Hum Mol Genet 2001 10 2961 2972 10.1093/hmg/10.26.2961 11751678

[B6] Zlotogora J Carmi R Lev B Shalev SA A targeted population carrier screening program for severe and frequent genetic diseases in Israel Eur J Hum Genet 2009 17 591 597 10.1038/ejhg.2008.241 19107146 PMC2986253

[B7] Laberge AM Michaud J Richter A Lemyre E Lambert M Brais B Mitchell GA Population history and its impact on medical genetics in Quebec Clin Genet 2005 68 287 301 10.1111/j.1399-0004.2005.00497.x 16143014

[B8] Sandron F La baisse de la fécondité en Tunisie. Centre Français sur la population et le développement (CEPED) 1998 Juillet, Paris

[B9] Institut National de la Statistique http://www.ins.nat.tn/fr/serie_annuelle_theme.php?code_theme=0201

[B10] The Fund for Peacehttp://www.fundforpeace.org/global/?q=states-tunisia

[B11] Riou S el Younsi C Chaabouni H Consanguinity in the population of northern Tunisia Tunis Med 1989 67 167 172 2756579

[B12] Kerkeni E Monastiri K Saket B Guediche MN Ben Cheikh H Interplay of socio-economic factors, consanguinity, fertility, and offspring mortality in Monastir, Tunisia Croat Med J 2007 48 701 707 17948956 PMC2205975

[B13] Kerkeni E Monastiri K Saket B Rudan D Zgaga L Ben Cheikh H Association among education level, occupation status, and consanguinity in Tunisia and Croatia Croat Med J 2006 47 656 661 16912991 PMC2080442

[B14] Ben Arab S Masmoudi S Beltaief N Hachicha S Ayadi H Consanguinity and endogamy in Northern Tunisia and its impact on non-syndromic deafness Genet Epidemiol 2004 27 74 79 10.1002/gepi.10321 15185405

[B15] Human Development Reports Statistics http://hdrstats.undp.org/2008/countries/country_fact_sheets/cty_fs_TUN.html

[B16] Seklani M La population de la Tunisie 1975 Paris

[B17] Camps G Les civilisations préhistoriques de l'Afrique du Nord et du Sahara 1974 Drouin Edition, Paris

[B18] Camps G Les Berbères. Mémoire et identité 1989 Errance Edition, Paris

[B19] Abun-Nasr AJM History of the Maghrib 1975 2 Cambridge University Press, Cambridge

[B20] Tabutin D Vilquin E Biraben JN L'histoire de la population de l'Afrique du Nord pendant le deuxième millénaire 2002 Université catholique de Louvain, Département des sciences de la population et du développement, Louvain-la-Neuve

[B21] Guyon JLG Histoire chronologique des épidémies du nord de l’Afrique 1855 Imprimerie du gouvernement, Alger

[B22] Jewish virtual library http://www.jewishvirtuallibrary.org/jsource/vjw/Tunisiavjw.html

[B23] Behar DM Metspalu E Kivisild T Rosset S Tzur S Hadid Y Yudkovsky G Rosengarten D Pereira L Amorim A Kutuev I Gurwitz D Bonne-Tamir B Villems R Skorecki K Counting the Founders: The Matrilineal Genetic Ancestry of the Jewish Diaspora PLoS One 2008 3 e2062 10.1371/journal.pone.0002062 18446216 PMC2323359

[B24] Manni F Leonardi P Patin E Berrebi A Khodjet El Khil H Skorecki K Rosengarten D Rouba H Heyer E Fellous M A Y-chromosome portrait of the population of Jerba (Tunisia) to elucidate its complex demographic history. Bulletins et Mémoires de la Société d 2005 Tome 17, Fascicule, Anthropologie de Paris 1

[B25] Cherni L Loueslati Yaâcoubi B Pereira L Alves C Khodjet-El-Khil H Ben Ammar El Gaaied A Amorim A Data for 15 autosomal STR markers (Powerplex 16 System) from two Tunisian populations: Kesra (Berber) and Zriba (Arab) Forensic Sci Int 2005 147 101 106 10.1016/j.forsciint.2004.04.009 15541599

[B26] Loueslati BY Cherni L Khodjet-Elkhil H Ennafaa H Pereira L Amorim A Ben Ayed F Ben Ammar Elgaaied A Islands inside an island: reproductive isolates on Jerba island Am J Hum Biol 2006 18 149 153 10.1002/ajhb.20473 16378336

[B27] Frigi S Pereira F Pereira L Yacoubi B Gusmão L Alves C Khodjet El Khil H Cherni L Amorim A El Gaaied A Data for Y-chromosome haplotypes defined by 17 STRs [AmpFLSTR Yfiler] in two Tunisian Berber communities Forensic Sci Int 2006 160 80 83 10.1016/j.forsciint.2005.05.007 16005592

[B28] Bahri R Esteban E Moral P Chaabani H New insights into the genetic history of Tunisians: data from Alu insertion and apolipoprotein E gene polymorphisms Ann Hum Biol 2008 35 22 33. 29 10.1080/03014460701753729 18274923

[B29] Hajjej A Kâabi H Sellami MH Dridi A El Borgi W Cherif G Elgaâïed A Almawi WY Boukef K Hmida S The contribution of HLA class I and II alleles and haplotypes to the investigation of the evolutionary history of Tunisians Tissue Antigens 2006 68 153 162 30 10.1111/j.1399-0039.2006.00622.x 16866885

[B30] El Moncer W Esteban E Bahri R Gayà-Vidal M Carreras-Torres R Athanasiadis G Moral P Chaabani H Mixed origin of the current Tunisian population from the analysis of Alu and Alu/STR compound systems J Hum Genet 2010 55 827 833 10.1038/jhg.2010.120 20882034

[B31] Mutalyzer Mutalyzerhttp://www.mutalyzer.nl/2.0/index

[B32] Tamura D DiGiovanna JJ Kraemer KH Founder mutations in xeroderma pigmentosum J Invest Dermatol 2010 130 1491 1493 10.1038/jid.2010.76 20463673 PMC3486739

[B33] Jelassi A Jguirim I Najah M Maatouk F Ben Hamda K Slimane MN Familial hypercholesterolemia in Tunisia Pathol Biol [Paris] 2009 57 444 450 10.1016/j.patbio.2008.09.015 19041195

[B34] Elloumi-Zghal H Barbouche MR Chemli J Béjaoui M Harbi A Snoussi N Abdelhak S Dellagi K Clinical and Genetic Heterogeneity of Inherited Autosomal Recessive Susceptibility to Disseminated Mycobacterium bovis Bacille Calmette-Guérin Infection J Infect Dis 2002 185 1468 1475 10.1086/340510 11992283

[B35] Tamary H Bar-Yam R Shalmon L Rachavi G Krostichevsky M Elhasid R Barak Y Kapelushnik J Yaniv I Auerbach AD Zaizov R Fanconi anaemia group A [FANCA] mutations in Israeli non-Ashkenazi Jewish patients Br J Haematol 2000 111 338 343 10.1046/j.1365-2141.2000.02323.x 11091222

[B36] Segal A Zivelin A Rosenberg N Ginsburg D Shpilberg O Seligsohn U A mutation in LMAN1 (ERGIC-53) causing combined factor V and factor VIII deficiency is prevalent in Jews originating from the island of Djerba in Tunisia Blood Coagul Fibrinolysis 2004 15 99 102 10.1097/00001721-200401000-00016 15166951

[B37] Marrakchi S Audebert S Bouadjar B Has C Lefèvre C Munro C Cure S Jobard F Morlot S Hohl D Prud'homme JF Zahaf A Turki H Fischer J Novel mutations in the gene encoding secreted lymphocyte antigen-6/urokinase-type plasminogen activator receptor-related protein-1 (SLURP-1) and description of five ancestral haplotypes in patients with Mal de Meleda J Invest Dermatol 2003 120 351 355 10.1046/j.1523-1747.2003.12062.x 12603845

[B38] Roume J Genin E Cormier-Daire V Ma HW Mehaye B Attie T Razavi-Encha F Fallet-Bianco C Buenerd A Clerget-Darpoux F Munnich A Le Merrer M A gene for Meckel syndrome maps to chromosome 11q13 Am J Hum Genet 1998 63 1095 1101 10.1086/302062 9758620 PMC1377494

[B39] Messaoud O Ben Rekaya M Cherif W Talmoudi F Boussen H Mokhtar I Boubaker S Amouri A Abdelhak S Zghal M Genetic homogeneity of mutational spectrum of group-A xeroderma pigmentosum in Tunisian patients Int J Dermatol 2010 49 544 548 10.1111/j.1365-4632.2010.04421.x 20534089

[B40] Haj Khelil A Denden S Leban N Daimi H Lakhdhar R Lefranc G Ben Chibani J Perrin P Hemoglobinopathies in North Africa: a review Hemoglobin 2010 34 1 23 10.3109/03630260903571286 20113284

[B41] Baert-Desurmont S Rouquette A Mauillon J Bouvignies E Soufir N Ratbi I Sefiani A Chaabouni H Frebourg T Une mutation à effet fondateur du gène MYH associée à la polypose adénomateuse et au cancer colorectal dans les pays du Maghreb. Société Nationale Française de Gastro-Entérologie 2007 http://www.snfge.asso.fr/01-Bibliotheque/0A-Resumes-JFHOD/2007/2061.htm

[B42] Brahimi N Jambou M Sarzi E Serre V Boddaert N Romano S de Lonlay P Slama A Munnich A Rötig A Bonnefont JP Lebre AS The first founder DGUOK mutation associated with hepatocerebral mitochondrial DNA depletion syndrome Mol Genet Metab 2009 97 221 226 10.1016/j.ymgme.2009.03.007 19394258

[B43] Hanein S Perrault I Roche O Gerber S Khadom N Rio M Boddaert N Jean-Pierre M Brahimi N Serre V Chretien D Delphin N Fares-Taie L Lachheb S Rotig A Meire F Munnich A Dufier JL Kaplan J Rozet JM TMEM126A, encoding a mitochondrial protein, is mutated in autosomal-recessive nonsyndromic optic atrophy Am J Hum Genet 2009 84 493 498 10.1016/j.ajhg.2009.03.003 19327736 PMC2667974

[B44] Wiszniewski W Fondaneche MC Lambert N Masternak K Picard C Notarangelo L Schwartz K Bal J Reith W Alcaide C de Saint Basile G Fischer A Lisowska-Grospierre B Founder effect for a 26-bp deletion in the RFXANK gene in North African major histocompatibility complex class II-deficient patients belonging to complementation group B Immunogenetics 2000 51 261 267 10.1007/s002510050619 10803838

[B45] Richard P Gaudon K Haddad H Ammar AB Genin E Bauché S Paturneau-Jouas M Müller JS Lochmüller H Grid D Hamri A Nouioua S Tazir M Mayer M Desnuelle C Barois A Chabrol B Pouget J Koenig J Gouider-Khouja N Hentati F Eymard B Hantaï D The CHRNE 1293insG founder mutation is a frequent cause of congenital myasthenia in North Africa Neurology 2008 71 1967 1972 10.1212/01.wnl.0000336921.51639.0b 19064877

[B46] Soufir N Ged C Bourillon A Austerlitz F Chemin C Stary A Armier J Pham D Khadir K Roume J Hadj-Rabia S Bouadjar B Taieb A de Verneuil H Benchiki H Grandchamp B Sarasin A A prevalent mutation with founder effect in xeroderma pigmentosum group C from North Africa J Invest Dermatol 2010 130 1537 1542 10.1038/jid.2009.409 20054342

[B47] Fathallah DM Bejaoui M Lepaslier D Chater K Sly WS Dellagi K Carbonic anhydrase II [CA II] deficiency in Maghrebian patients: evidence for founder effect and genomic recombination at the CA II locus Hum Genet 1997 99 634 637 10.1007/s004390050419 9150731

[B48] Petit FM Bézieau S Gajdos V Parisot F Scoul C Capel L Stozinic V Khrouf N M'Rad R Koshy A Mollet-Boudjemline A Francoual J Labrune P The Tunisian population history through the Crigler-Najjar type I syndrome Eur J Hum Genet 2008 16 848 853 10.1038/sj.ejhg.5201989 18197191

[B49] Ouahchi K Arita M Kayden H Hentati F Ben Hamida M Sokol R Arai H Inoue K Mandel JL Koenig M Ataxia with isolated vitamin E deficiency is caused by mutations in the alpha-tocopherol transfer protein Nat Genet 1995 9 141 145 10.1038/ng0295-141 7719340

[B50] Bouchlaka C Maktouf C Mahjoub B Ayadi A Sfar MT Sioud M Gueddich N Belhadjali Z Rebaï A Abdelhak S Dellagi K Genetic heterogeneity of megaloblastic anaemia type 1 in Tunisian patients J Hum Genet 2007 52 262 270 10.1007/s10038-007-0110-0 17285242

[B51] Tullio-Pelet A Salomon R Hadj-Rabia S Mugnier C de Laet MH Chaouachi B Bakiri F Brottier P Cattolico L Penet C Bégeot M Naville D Nicolino M Chaussain JL Weissenbach J Munnich A Lyonnet S Mutant WD-repeat protein in triple-A syndrome Nat Genet 2000 263 332 335 11062474 10.1038/81642

[B52] Warren L Gibson R Ishihara L Elango R Xue Z Akkari A Ragone L Pahwa R Jankovic J Nance M Freeman A Watts RL Hentati F A founding LRRK2 haplotype shared by Tunisian, US, European and Middle Eastern families with Parkinson's disease Parkinsonism Relat Disord 2008 14 77 80 10.1016/j.parkreldis.2007.02.001 17433753

[B53] Gilad S Bar-Shira A Harnik R Shkedy D Ziv Y Khosravi R Brown K Vanagaite L Xu G Frydman M Lavin MF Hill D Tagle DA Shiloh Y Ataxia-telangiectasia: founder effect among North African Jews Hum Mol Genet 1996 5 2033 2037 10.1093/hmg/5.12.2033 8968760

[B54] Falik-Zaccai TC Shachak E Yalon M Lis Z Borochowitz Z Macpherson JN Nelson DL Eichler EE Predisposition to the fragile X syndrome in Jews of Tunisian descent is due to the absence of AGG interruptions on a rare Mediterranean haplotype Am J Hum Genet 1997 60 103 112 8981953 PMC1712540

[B55] Yakobson E Eisenberg S Isacson R Halle D Levy-Lahad E Catane R Safro M Sobolev V Huot T Peters G Ruiz A Malvehy J Puig S Chompret A Avril MF Shafir R Peretz H Bressac-de Paillerets B A single Mediterranean, possibly Jewish, origin for the Val59Gly CDKN2A mutation in four melanoma-prone families Eur J Hum Genet 2003 11 288 296 10.1038/sj.ejhg.5200961 12700603

[B56] Touitou I The spectrum of Familial Mediterranean Fever (FMF) mutations Eur J Hum Genet 2001 9 473 483 10.1038/sj.ejhg.5200658 11464238

[B57] Tishkoff SA Varkonyi R Cahinhinan N Abbes S Argyropoulos G Destro-Bisol G Drousiotou A Dangerfield B Lefranc G Loiselet J Piro A Stoneking M Tagarelli A Tagarelli G Touma EH Williams SM Clark AG Haplotype diversity and linkage disequilibrium at Human G6PD: Recent origin of alleles that confer malarial resistance Science 2002 293 445 462 10.1126/science.1061573 11423617

[B58] Kharrat M Tardy V M'Rad R Maazoul F Jemaa LB Refaï M Morel Y Chaabouni H Molecular genetic analysis of tunisian patients with classic form of 21-Hydroxylase Deficiency: Identification of four novel mutations and high prevalence of Q318X mutation J Clin Endocrinol Metab 2004 89 368 374 10.1210/jc.2003-031056 14715874

[B59] Abid F Tardy V Gaouzi A El Hessni A Morel Y Chabraoui L CYP21A2 gene mutation analysis in Moroccan patients with classic form of 21-hydroxylase deficiency: high regional prevalence of p.Q318X mutation and identification of a novel p.L353R mutation Clin Chem Lab Med 2008 4612 1707 1713 18973462 10.1515/CCLM.2008.339

[B60] Kefi M Amouri R Driss A Ben Hamida C Ben Hamida M Kunkel LM Hentati F Phenotype and sarcoglycan expression in Tunisian LGMD 2C patients sharing the same del521-T mutation Neuromuscul Disord 2003 13 779 787 10.1016/S0960-8966(03)00136-6 14678800

[B61] Belguith H Hajji S Salem N Charfeddine I Lahmar I Amor MB Ouldim K Chouery E Driss N Drira M Mégarbané A Rebai A Sefiani A Masmoudi S Ayadi H Analysis of GJB2 mutation: evidence for a Mediterranean ancestor for the 35delG mutation Clin Genet 2005 68 188 189 15996220 10.1111/j.1399-0004.2005.00474.x

[B62] Kharrat M Trabelsi S Chaabouni M Maazoul F Kraoua L Ben Jemaa L Gandoura N Barsaoui S Morel Y M'rad R Chaabouni H Only two mutations detected in 15 Tunisian patients with 11beta-hydroxylase deficiency: the p.Q356X and the novel p.G379V Clin Genet 2010 78 398 4011 10.1111/j.1399-0004.2010.01403.x 20331679

[B63] Zhu YS Cordero JJ Can S Cai LQ You X Herrera C DeFillo-Ricart M Shackleton C Imperato-McGinley J Mutations in CYP11B1 Gene: Phenotype–Genotype Correlations Am J Med Genet A 2003 122A 193 200 10.1002/ajmg.a.20108 12966519

[B64] Troudi W Uhrhammer N Romdhane KB Sibille C Amor MB Khodjet El Khil H Jalabert T Mahfoudh W Chouchane L Ayed FB Bignon YJ Elgaaied AB Complete mutation screening and haplotype characterization of BRCA1 gene in Tunisian patients with familial breast cancer Cancer Biomark 2008 4 11 18 18334730 10.3233/cbm-2008-4102

[B65] Cherbal F Bakour R Adane S Boualga K Benais-Pont G Maillet P BRCA1 and BRCA2 germline mutations screening in Algerian breast/ovarian cancer families Dis Markers 2010 28 377 384 20683152 10.3233/DMA-2010-0718 PMC3833328

[B66] Messaoud T Bel Haj Fredj S Bibi A Elion J Férec C Fattoum S Molecular epidemiology of cystic fibrosis in Tunisia Ann Biol Clin [Paris] 2005 63 627 630 16330381

[B67] Lakeman P Gille JJ Dankert-Roelse JE Heijerman HG Munck A Iron A Grasemann H Schuster A Cornel MC Ten Kate LP CFTR mutations in Turkish and North African cystic fibrosis patients in Europe: implications for screening Genet Test 2008 12 25 35 10.1089/gte.2007.0046 18373402

[B68] Mili A Ben Charfeddine I Mamaï O Abdelhak S Adala L Amara A Pagliarani S Lucchiarri S Ayadi A Tebib N Harbi A Bouguila J H'mida D Saad A Limem K Comi GP Gribaa M Molecular and biochemical characterization of Tunisian patients with glycogen storage disease type III J Hum Genet 2012 53 170 175 22089644 10.1038/jhg.2011.122

[B69] Endo Y Horinishi A Vorgerd M Aoyama Y Ebara T Murase T Odawara M Podskarbi T Shin YS Okubo M Molecular analysis of the AGL gene: heterogeneity of mutations in patients with glycogen storage disease type III from Germany, Canada, Afghanistan, Iran, and Turkey J Hum Genet 2006 51 958 963 10.1007/s10038-006-0045-x 17047887

[B70] Perrault I Rozet JM Gerber S Ghazi I Ducroq D Souied E Leowski C Bonnemaison M Dufier JL Munnich A Kaplan J Spectrum of retGC1 mutations in Leber's congenital amaurosis Eur J Hum Genet 2000 8 578 582 10.1038/sj.ejhg.5200503 10951519

[B71] Ben Mustapha I Kammoun A Mellouli F Abdelmoula S Chemli J Largueche B Riahi R Bejaoui M Barbouche MR A strong founder effect for a 10-bp deletion in the CD18 gene in North African leukocyte adhesion deficiency type 1 patients Clin Exp Immunol 2008 154 Suppl1 105

[B72] Fathallah DM Jamal T Barbouche MR Bejaoui M Hariz MB Dellagi K Two Novel Frame Shift, recurrent and De Novo Mutations in the ITGB2 CD18 Gene Causing Leukocyte Adhesion Deficiency in a Highly Inbred North African Population J Biomed Biotechnol 2001 13 114 121 12488604 10.1155/S1110724301000250 PMC129056

[B73] Charfeddine C Mokni M Ben Mousli R Elkares R Bouchlaka C Boubaker S Ghedamsi S Baccouche D Ben Osman A Dellagi K Abdelhak S A novel missense mutation in the gene encoding SLURP-1 in patients with Mal de Meleda from northern Tunisia Br J Dermatol 2003 149 1108 1115 10.1111/j.1365-2133.2003.05606.x 14674887

[B74] Smaoui N Chaabouni M Sergeev YV Kallel H Li S Mahfoudh N Maazoul F Kammoun H Gandoura N Bouaziz A Nouiri E M'Rad R Chaabouni H Hejtmancik JF Screening of the eight BBS genes in Tunisian families: no evidence of triallelism Invest Ophthalmol Vis Sci 2006 47 3487 3495 10.1167/iovs.05-1334 16877420

[B75] Zlotogora J High frequencies of human genetic diseases: founder effect with genetic drift or selection? Am J Med Genet 1994 49 10 13 10.1002/ajmg.1320490104 8172234

[B76] Feingold J Multiple mutations in a specific gene in a small population C R Acad Sci III 1998 321 553 555 10.1016/S0764-4469(98)80456-3 9769854

[B77] Noguchi S McNally EM Ben Othmane K Hagiwara Y Mizuno Y Yoshida M Yamamoto H Bönnemann CG Gussoni E Denton PH Kyriakides T Middleton L Hentati F Ben Hamida M Nonaka I Vance JM Kunkel LM Ozawa E Mutations in the dystrophin-associated protein gamma-sarcoglycan in chromosome 13 muscular dystrophy Science 1995 270 819 822 10.1126/science.270.5237.819 7481775

[B78] Hentati F Muscular dystrophies in Arab countries 2008 The First Educational Workshop in Developing Countries on Genetic Counseling, Beirut, Lebanon http://www.jeans4genes.org/files/GC_arab_states.pdf

[B79] Messaoud O Ben Rekaya M Ouragini H Benfadhel S Azaiez H Kefi R Gouider-Khouja N Mokhtar I Amouri A Boubaker MS Zghal M Abdelhak S Severe phenotypes in two Tunisian families with novel XPA mutations: evidence for a correlation between mutation location and disease severity Arch Dermatol Res 2012 304 171 176 10.1007/s00403-011-1190-4 22081045

[B80] Chaabouni HB Ksantini M M'rad R Kharrat M Chaabouni M Maazoul F Bahloul Z Ben Jemaa L Ben Moussa F Ben Chaabane T Mrad S Touitou I Smaoui N MEFV mutations in Tunisian patients suffering from familial Mediterranean fever Semin Arthritis Rheum 2007 36 397 401 10.1016/j.semarthrit.2006.12.004 17276496

[B81] Zlotogora J Multiple mutations responsible for frequent genetic diseases in isolated populations Eur J Hum Genet 2007 15 272 278 10.1038/sj.ejhg.5201760 17213840

[B82] Kharfi M El Fékih N Aounallah-Skhiri H Schmitt S Fazaa B Küry S Kamoun MR Acrodermatitis enteropathica: a review of 29 Tunisian cases Int J Dermatol 2010 49 1038 1044 10.1111/j.1365-4632.2010.04566.x 20883266

[B83] Almaani N Liu L Dopping-Hepenstal PJ Lai-Cheong JE Wong A Nanda A Moss C Martinéz AE Mellerio JE McGrath JA Identical Glycine Substitution Mutations in Type VII Collagen May Underlie Both Dominant and Recessive Forms of Dystrophic Epidermolysis Bullosa Acta Derm Venereol 2011 91 262 266 10.2340/00015555-1053 21448560

[B84] Van Laer L Coucke P Mueller RF Caethoven G Flothmann K Prasad SD Chamberlin GP Houseman M Taylor GR Van de Heyning CM Fransen E Rowland J Cucci RA Smith RJ Van Camp G A common founder for the 35delG GJB2 gene mutation in connexin 26 hearing impairment J Med Genet 2001 38 515 518 10.1136/jmg.38.8.515 11483639 PMC1734914

[B85] Kokotas H Grigoriadou M Villamar M Giannoulia-Karantana A del Castillo I Petersen MB Hypothesizing an ancient Greek origin of the GJB2 35delG mutation: can science meet history? Genet Test Mol Biomarkers 2010 14 183 187 10.1089/gtmb.2009.0146 20073550

[B86] Kokotas H Van Laer L Grigoriadou M Iliadou V Economides J Pomoni S Pampanos A Eleftheriades N Ferekidou E Korres S Giannoulia-Karantana A Van Camp G Petersen MB Strong linkage disequilibrium for the frequent GJB2 35delG mutation in the Greek population Am J Med Genet A 2008 146A 2879 2884 10.1002/ajmg.a.32546 18925674

[B87] Byers P Age and Origin of the PRNP E200K Mutation Causing Familial Creutzfeldt-Jacob Disease in Libyan Jews Am J Hum Genet 2000 67 528 531 10.1086/303021 10889050 PMC1287202

[B88] Zlotogora J Knowing the ethnic origin of the patient is important in making a diagnosis Am J Med Genet 1998 78 393 394 10.1002/(SICI)1096-8628(19980724)78:4<393::AID-AJMG23>3.0.CO;2-C 9714449

[B89] Ben Hamida M Fardeau M Attia N Severe childhood muscular dystrophy affecting both sexes and frequent in Tunisia Muscle Nerve 1983 6 469 480 10.1002/mus.880060702 6633560

[B90] Civeira F Ros E Jarauta E Plana N Zambon D Puzo J de Esteban JP M Ferrando J Zabala S Almagro F Gimeno JA Masana L Pocovi M Comparison of genetic versus clinical diagnosis in familial hypercholesterolemia Am J Cardiol 2008 102 1187 1193 10.1016/j.amjcard.2008.06.056 18940289

[B91] Slimane MN Pousse H Maatoug F Hammami M BenFarhat MH Phenotypic expression of familial hypercholesterolemia in central and southern Tunisia Atherosclerosis 1993 104 153 158 10.1016/0021-9150(93)90186-X 8141839

[B92] Bujo H Takahashi K Saito Y Maruyama T Yamashita S Matsuzawa Y Ishibashi S Shionoiri F Yamada N Kita T Research Committeon Primary Hyperlipidemia of the Ministry of Health, Labour, and Welfare of Japan. Clinical features of familial hypercholesterolemia in Japan in a database from 1996–1998 by the research committee of the ministry of health, labour and welfare of Japan J Atheroscler Thromb 2004 11 146 151 10.5551/jat.11.146 15256765

[B93] Wu LL Hopkins PN Xin Y Stephenson SH Williams RR Nobe Y Kajita M Nakajima T Emi M Co-segregation of elevated LDL with a novel mutation [D92K] of the LDL receptor in a kindred with multiple lipoprotein abnormalities J Hum Genet 2000 45 154 158 10.1007/s100380050202 10807540

[B94] Civeira F Jarauta E Cenarro A García-Otín AL Tejedor D Zambón D Mallen M Ros E Pocoví M Frequency of Low-Density Lipoprotein Receptor Gene Mutations in Patients with a Clinical Diagnosis of Familial Combined Hyperlipidemia in a Clinical Setting J Am Coll Cardiol 2008 52 1546 1553 10.1016/j.jacc.2008.06.050 19007590

[B95] Goldstein JL Schrott HG Hazzard WR Bierman EL Motulsky AG Hyperlipidemia in coronary heart disease. II. Genetic analysis of lipid levels in 176 families and delineation of a new inherited disorder, combined hyperlipidemia J Clin Invest 1973 52 1544 1568 10.1172/JCI107332 4718953 PMC302426

[B96] Barkaoui E Cherif W Tebib N Charfeddine C Ben Rhouma F Azzouz H Ben Chehida A Monastiri K Chemli J Amri F Ben Turkia H Abdelmoula MS Kaabachi N Abdelhak S Ben Dridi MF Mutation spectrum of glycogen storage disease type Ia in Tunisia: Implication for molecular diagnosis J Inherit Metab Dis 2007 30 989 10.1007/s10545-007-0737-1 18008183

[B97] Ben Rekaya M Messaoud O Talmoudi F Nouira S Ouragini H Amouri A Boussen H Boubaker S Mokni M Mokthar I Abdelhak S Zghal M High frequency of the V548AfsX572 XPC mutation in Tunisia: implication for molecular diagnosis J Hum Genet 2009 54 426 429 10.1038/jhg.2009.50 19478817

[B98] Khan SG Oh KS Shahlavi T Ueda T Busch DB Inui H Emmert S Imoto K Muniz-Medina V Baker CC DiGiovanna JJ Schmidt D Khadavi A Metin A Gozukara E Slor H Sarasin A Kraemer KH Reduced XPC DNA repair gene mRNA levels in clinically normal parents of xeroderma pigmentosum patients Carcinogenesis 2006 27 84 94 16081512 10.1093/carcin/bgi204

[B99] Chkioua L Khedhiri S Turkia HB Tcheng R Froissart R Chahed H Ferchichi S Ben Dridi MF Vianey-Saban C Laradi S Miled A Mucopolysaccharidosis type I: identification of alpha-L-iduronidase mutations in Tunisian families Arch Pediatr 2007 14 1183 1189 10.1016/j.arcped.2007.06.018 17728118

[B100] Alif N Hess K Straczek J Sebbar S N'Bou A Nabet P Dousset B Mucopolysaccharidosis type I: characterization of a common mutation that causes Hurler syndrome in Moroccan subjects Ann Hum Genet 1999 63 9 16 10.1046/j.1469-1809.1999.6310009.x 10738517

